# Socioeconomic and Demographic Factors in Genetic Testing Utilization Among Advanced Prostate Cancer Patients

**DOI:** 10.1002/pros.70165

**Published:** 2026-03-30

**Authors:** Alexandra T. Skowron, Karen Childers, Courtney M. Rose, Kyle C. McElyea, Avery K. Supernois, Yousuf Hussain, Mohammed Baseer, Sunita Ghosh, Clara Hwang

**Affiliations:** ^1^ Wayne State University School of Medicine Detroit Michigan USA; ^2^ Department of Internal Medicine Division of Hematology and Oncology, Henry Ford Health Detroit Michigan USA; ^3^ Department of Public Health Sciences Division of Biostatistics, Henry Ford Health Detroit Michigan USA; ^4^ Department of Public Health Sciences Henry Ford Health Detroit Michigan USA; ^5^ Michigan State University Lansing Michigan USA

**Keywords:** barriers, disparities, genetic testing, prostate cancer, social determinants of health

## Abstract

**Purpose:**

Germline genetic testing in patients with advanced prostate cancer (PCa) is underutilized and hypothesized to be impacted by socioeconomic and demographic factors. This single institution, retrospective study assessed the association of income and social vulnerability with genetic referrals and testing.

**Methods:**

The Henry Ford Health (HFH) tumor registry was queried for new diagnoses of stage III, IV PCa from 2017 to 2022. 1385 patients were eligible and 186 patients (13.43%) received a referral, while 1199 (86.57%) patients did not. Median household income (gMHI) and social vulnerability index (SVI) were assigned based on census tract level geocoding and analyzed by tertiles. Univariable and multivariable logistic regression analyses were performed assessing referral for genetic counseling and completion of genetic testing as outcomes. In the multivariable analysis, associations between age, stage and gMHI were analyzed.

**Results:**

Black and stage IV patients were more likely to receive genetics referral than White and stage III patients, respectively (20.3% vs 10.6%, *p* < 0.0001; 25.5% vs 7.3%, *p* < 0.0001). Multivariable analysis showed that middle gMHI tertile patients had 53% lower unadjusted OR for genetic referral than lowest gMHI tertile patients (OR = 0.47, 95% CI [0.29, 0.75]; *p* = 0.0017). There was no significant difference in the odds of referral between the highest and lowest gMHI tertiles (OR = 1.22, 95% CI [0.83, 1.79]; *p* = 0.3175). No other significant associations were found between gMHI, SVI and genetic counseling referrals or completion of testing. Adjusted comparisons showed significant associations between increased referral or testing rate and decreasing age and more advanced stage (OR = 0.97, 95% CI [0.95, 1.00]; *p* = 0.0218), (OR = 2.84, 95% CI [1.70, 4.74]; *p* = 0.0001).

**Conclusion:**

Although socioeconomic factors have been hypothesized as barriers to genetic testing, we found no evidence of an association in our patient population. Instead, the lowest gMHI tertile patients were equally as likely to complete genetic testing as highest gMHI tertile patients, indicating socioeconomic barriers can be overcome. Clinical factors including age and stage did independently predict completion of testing, supporting referral for testing based on clinical characteristics.

## 1 Introduction

Currently, germline genetic testing in patients with advanced prostate cancer (PCa) is underutilized, despite being recommended in NCCN guidelines [1, 2, 3]. A 2023 study from Aguiar et al. demonstrated that less than 10% of PCa patients with localized disease and only 35.5% of patients with metastatic disease completed germline genetic testing [4]. Current research estimates that 5%–15% of PCa can be attributed to hereditary factors such as mutations in mismatch repair genes (*MLH1*, *MSH2*, *MSH6,* and *PMS2*) and homologous recombination genes (*BRCA1, BRCA2, ATM, CHEK2)* [5]. Targeted therapies, such as pembrolizumab for mismatch‐repair‐deficient tumors or poly (ADP‐ribose) polymerase (PARP) inhibitors for homologous recombination repair‐deficient tumors, may improve outcomes for patients with those mutations [5, 6]. Additionally, germline testing can impact family care, providing insights into increased cancer risk for family members and allowing for potential prevention and earlier intervention. Therefore, it is important to probe patient and provider data to better understand why germline genetic testing is not being utilized more frequently.

Several research studies over the past few years have explored physician attitudes and practices towards germline genetic testing for PCa patients [1, 2, 3]. In one such study by Paller et al., only 62% of genitourinary (GU) oncologists reported considering genetic testing for all metastatic PCa patients, citing barriers such as limited access to counseling, lack of insurance coverage and insufficient education materials [1]. Similarly, Loeb et al. found that physician knowledge and concerns about cost were also primary barriers to implementing germline genetic testing for PCa patients [2]. Results from Gunn et al. echoed these findings and added the additional barrier of inaccessible or inaccurate family history [3]. Many of these barriers extend beyond household income and cost of care into accessibility and access. To address social vulnerability more wholistically, the CDC developed a tool called the social vulnerability index (SVI) [7]. SVI uses 16 US Census variables such as socioeconomic status, housing type and transportation, and racial and ethnic minority status, to identify communities that may need extra support [7]. It is reported on a scale of 0–1 with higher values corresponding to more socially vulnerable populations [7]. These socioeconomic factors could be preventing lower‐income or more socially vulnerable PCa patients from receiving the genetic testing that they need to better their care or educate their families on potential cancer risk in the future. In this single site, retrospective study, we aimed to further understand how these barriers may be affecting germline genetic testing for PCa patients. We hypothesized that lower geocoded neighborhood median household income (gMHI) and higher SVI would have an association with less access to and completion of genetic testing. We also analyzed whether these associations were independent of other clinical factors likely to predict genetic counseling referral or testing completion, such as age, family history, and stage. This analysis further expounded on findings from our lab group published in The Prostate in December 2025 [8].

## 2 Methods

### 2.1 Data Collection

Given that guidelines state that germline testing is recommended for patients with metastatic, regional (node positive), very‐high‐risk localized, or high‐risk localized PCa [9], the Henry Ford Cancer Registry (HFCR) and electronic medical record system, EPIC, were queried for new diagnoses of stage III, stage IVa and stage IVb PCa between 2017 and 2022. Per AJCC v8, stage III PCa is defined as localized disease (no nodal or distant metastases) with at least one of the following high‐risk features: extraprostatic extension that may involve seminal vesicles or adjacent pelvic organs, PSA ≥ 20, or Grade Group 5 disease. Stage IV PCa is a disease that has spread to lymph nodes or distant sites. Based on updates to NCCN guidelines, all patients with stage III and IV PCa are eligible for genetic referral. The HFCR is maintained by trained abstractors who extract data from initial oncologic consultations through follow up visits for 180 days. Diagnoses were staged in accordance with the American Joint Committee on Cancer (AJCC) eighth edition by trained abstractors, using both clinical and pathological data found in the HFCR. ECOG performance status was determined by the clinician at each clinic visit and subsequently tracked in HFCR. Data from patients with ECOG 4 status was not reviewed because genetic counseling consultation referrals and attendance are unlikely in the context of severe disability. Demographic information, such as race and ethnicity, was based on information provided by patients at their initial oncologic consultation.

Patient addresses were obtained at initial clinical visits and subsequently confirmed and tracked in HFCR. Census tract level, neighborhood‐based geocoding was then assigned to generate both Median household income (gMHI) and SVI data for each patient. Licensed versions of Pitney Bowes MapInfo and MapMarker software were used to generate this data. Patients' home addresses were accessed in February 2024 to generate these data points. SVI was reported on a scale of 0 to 1, with 1 being the least vulnerable and 0 being the most vulnerable.

Genetic testing outcomes were primarily manually abstracted from patient charts by trained abstractors. Referral information is available in EPIC Clarity, an SQL server‐based database that supports EPIC's clinical information system. Referrals analyzed were only those explicitly documented in patient charts. Any discussion of genetic testing between patient and provider that did not result in a documented referral or testing completion was not included in this analysis.

Outcomes were referral for genetic testing (yes vs. no) and completion of genetic testing (yes vs. no). Wilcoxon rank sum test was used to compare non‐normally distributed continuous variables between groups and the Chi‐squared test was used to compare the categorical variables. Univariable and multivariable logistic regression analyses were used to explore associations with referral for genetic testing and completion of genetic testing.

### 2.2 Statistical Analysis

All continuous data were reported using median and IQR; categorical data were reported using counts and column percentages (n(%)). To make two group comparisons (patients who received a genetic referral versus did not receive a genetic referral and patients who completed genetic testing versus did not complete genetic testing), the Wilcoxon rank sum test was used for the continuous variables and Chi‐squared test was used for categorical variables. To explore the correlations between age of diagnosis and SVI or gMHI, the Spearman correlations were reported, which accounts for non‐normally distributed data. The association between social determinants of health and race was assessed. gMHI and SVI were categorized into three groups using tertiles, stratified by race, and compared using Chi‐squared tests. Univariable and multivariable binary logistic regression analysis were used to explore the associations with referral for genetic testing (yes vs. no) and completion of genetic testing (yes vs. no). The final multivariable regression analysis included fixed effects of age, gMHI, and stage (referral) or substage (completion). Unadjusted and adjusted odds ratios with 95% Wald confidence intervals were reported. Statistical significance was set at *p* < 0.05. All analyses are performed using SAS 9.4 (SAS Institute Inc., Cary, NC, USA).

## 3 Results

A total of 1385 patients met eligibility criteria for this analysis. 186 patients (13.43%) received a referral, while 1199 (86.57%) patients did not. 918 (66.3%) had Stage III PCa, 467 had Stage IV PCa, with 162 (11.7%) Stage IVa patients and 305 (22.0%) Stage IVb patients.

### 3.1 Associations Between gMHI, SVI and Referral for Genetic Testing

We analyzed patient demographics, including age at diagnosis, race, ethnicity, family history of cancer, ECOG score and stage, as well as both gMHI and SVI to find associations between these factors and referral for genetic counseling. No statistically significant associations were observed between age at diagnosis, ethnicity, family history, ECOG score, and referral to genetics. A significant association between race and referral (*p* < 0.0001) was observed as 10.6% of White patients (*n* = 101), 20.3% of Black patients (*n* = 379), and 14.6% patients who identified as other (*n* = 6) received a referral. Stage and referral (*p* < 0.0001) also showed statistically significant association with Stage IV patients receiving more referrals than Stage III patients. Lastly, there was an association between gMHI and referral showing that fewer patients in the middle tertile received a referral (*p* = 0.0001) (Table 1). No significant association was observed between SVI and referral.

**Table 1:** Demographics/MHI/SVI for all patients by genetic referral.

		Received referral for genetic counseling		
	Total (*N* = 1385)	Yes (*N* = 186)	No (*N* = 1199)	*p*‐value
Age at diagnosis, median (IQR)	67.0 (62.0, 73.0)	66.0 (59.0, 73.0)	68.0 (62.0, 73.0)	0.0834^a^
Race, *n* (%)				< 0.0001^b^
White	949 (69.3%)	101 (10.6%)	848 (89.4%)	
Black	379 (27.7%)	77 (20.3%)	302 (79.7%)	
Other	41 (3.0%)	6 (14.6%)	35 (85.4%)	
Missing	16	2	14	
Ethnicity, *n* (%)				0.3924^b^
Hispanic/Latino	20 (1.5%)	4 (20.0%)	16 (80.0%)	
Not Hispanic/Latino	1327 (98.5%)	178 (13.4%)	1149 (86.6%)	
Missing	38	4	34	
Family history of cancer, *n* (%)				0.1725^b^
Yes	240 (50.6%)	45 (18.8%)	195 (81.3%)	
No	234 (49.4%)	33 (14.1%)	201 (85.9%)	
Missing	911	108	803	
ECOG score, *n* (%)				0.4311^b^
0	862 (77.6%)	121 (14.0%)	741 (86.0%)	
1	217 (19.5%)	38 (17.5%)	179 (82.5%)	
2 or 3	32 (2.9%)	5 (15.6%)	27 (84.4%)	
Missing	274	22	252	
Stage, *n* (%)				< 0.0001^b^
3	918 (66.3%)	67 (7.3%)	851 (92.7%)	
4	467 (33.7%)	119 (25.5%)	348 (74.5%)	
Median household income ($), n (%)				0.0001^b^
16,683–61,508	371 (33.4%)	57 (15.4%)	314 (84.6%)	
61,509–83,767	370 (33.3%)	29 (7.8%)	341 (92.2%)	
83,768–233,173	370 (33.3%)	67 (18.1%)	303 (81.9%)	
Missing	274	33	241	
Social vulnerability index (IQR), *n* (%)				0.1456^b^
0.54–0.99	378 (33.8%)	59 (15.6%)	319 (84.4%)	
0.24–0.54	368 (32.9%)	40 (10.9%)	328 (89.1%)	
0–0.24	373 (33.3%)	54 (14.5%)	319 (85.5%)	
Missing	266	33	233	

^a^ Wilcoxon rank sum *p*‐value.

^b^ Chi‐Square *p*‐value.

### 3.2 Associations Between gMHI, SVI and Genetic Testing Completion

Genetic testing completion data was only available for patients with Stage IV disease (*n* = 467); 159 (34%) patients completed genetic testing and 308 (66%) did not. Patient demographics including age at diagnosis, race, ethnicity, family history of cancer, ECOG, gMHI and SVI were not associated with completion of genetic testing. Substage and completion of testing showed strong significant association (*p* < 0.0001). 21.6% of substage IVa patients (*n* = 35), and 40.7% of Stage IVb disease (*n* = 124) completed genetic testing (Table 2).

**Table 2:** Demographics/MHI/SVI for stage IV patients by completion of genetic testing.

		Completed genetic testing		
	Total (*N* = 467)	Testing (*N* = 159)	No testing (*N* = 308)	*p*‐value
Substage, *n* (%)				< 0.0001^a^
IVa	162 (34.7%)	35 (21.6%)	127 (78.4%)	
IVb	305 (65.3%)	124 (40.7%)	181 (59.3%)	
Age at diagnosis, median (IQR)	69.0 (62.0, 75.0)	68.0 (61.0, 75.0)	69.0 (62.0, 75.5)	0.2653^b^
Race, *n* (%)				0.4518^a^
White	301 (64.9%)	98 (32.6%)	203 (67.4%)	
Black	150 (32.3%)	55 (36.7%)	95 (63.3%)	
Other	13 (2.8%)	6 (46.2%)	7 (53.8%)	
Missing	3	0	3	
Ethnicity, *n* (%)				0.2858^a^
Hispanic/Latino	15 (3.3%)	7 (46.7%)	8 (53.3%)	
Not Hispanic/Latino	446 (96.7%)	149 (33.4%)	297 (66.6%)	
Missing	6	3	3	
Family history of cancer, *n* (%)				0.2185^a^
Yes	90 (52.9%)	41 (45.6%)	49 (54.4%)	
No	80 (47.1%)	29 (36.3%)	51 (63.8%)	
Missing	297	89	208	
ECOG score, *n* (%)				0.0858^a^
0	216 (58.7%)	73 (33.8%)	143 (66.2%)	
1	130 (35.3%)	56 (43.1%)	74 (56.9%)	
2 or 3	22 (6.0%)	5 (22.7%)	17 (77.3%)	
Missing	99	25	74	
Median household income ($), *n* (%)				0.5412^a^
16,683–61,508	161 (43.8%)	55 (34.2%)	106 (65.8%)	
61,509–83,767	110 (29.9%)	37 (33.6%)	73 (66.4%)	
83,768–233,173	97 (26.4%)	39 (40.2%)	58 (59.8%)	
Missing	99	28	71	
Social vulnerability index (IQR), *n* (%)				0.0553^a^
0.54–0.99	165 (44.2%)	56 (33.9%)	109 (66.1%)	
0.24–0.54	107 (28.7%)	31 (29.0%)	76 (71.0%)	
0–0.24	101 (27.1%)	45 (44.6%)	56 (55.4%)	
Missing	94	27	67	

^a^ Chi‐Square *p*‐value.

^b^ Wilcoxon rank sum *p*‐value.

### 3.3 gMHI and SVI Association With Race

The association between gMHI and SVI with respect to race (Black vs. White) was also examined. Race data included 379 Black patients and 949 White patients. There was an association between gMHI and race (*p* < 0.0001) with Black patients more likely to have lower median household income when compared to White patients. The majority of Black patients (65.4%, *n* = 200) were in the lower gMHI group while the majority of White patients (79.0%, *n* = 602) were in the middle and higher gMHI groups. There was also an association between SVI and race (*p* < 0.0001) with Black patients more likely to live in higher vulnerability communities than White patients. The majority of Black patients (65.1%, *n* = 203) fell in the highest SVI group, while the majority of White patients belong to the middle and lower SVI groups (Table 3).

**Table 3:** Association between MHI/SVI and race.

		Race		
	Total (*N* = 1328)	Black (*N* = 379)	White (*N* = 949)	*p*‐value
Median household income ($), *n* (%)				< 0.0001^a^
16,683–61,508	360 (33.7%)	200 (65.4%)	160 (21.0%)	
61,509–83,767	360 (33.7%)	50 (16.3%)	310 (40.7%)	
83,768–233,173	348 (32.6%)	56 (18.3%)	292 (38.3%)	
Missing	260	73	187	
Social vulnerability index (IQR), *n* (%)				< 0.0001^a^
0.55–0.99	364 (33.9%)	203 (65.1%)	161 (21.1%)	
0.25–0.54	356 (33.1%)	72 (23.1%)	284 (37.2%)	
0–0.24	355 (33.0%)	37 (11.9%)	318 (41.7%)	
Missing	253	67	186	

^a^ Chi‐square *p*‐value.

### 3.4 Probability of Predicting Referral for Genetic Counseling Adjusted for Age, Stage and gMHI

Using univariable and multivariable logistic regression models, we aimed to assess the efficacy of predicting referral for genetic counseling for age, stage and gMHI (Table 4). Marginal significant associations were observed between age and referral for genetic counseling for the unadjusted analysis (OR = 0.98, 95% CI [0.96, 1.00], *p* < 0.0469). When controlling for stage and gMHI, the odds of referral decreased by 3% for each 1‐year increase in age (OR = 0.97, 95% CI [0.95, 0.99]; *p* = 0.0052).

**Table 4:** Unadjusted and adjusted odds ratios predicting referral for genetic testing.

	Unadjusted OR [95% CI]^a^	Unadjusted *p*‐value	Adjusted OR [95% CI]^b^	Adjusted *p*‐value
Age	0.98 [0.96, 1.00]	0.0469	0.97 [0.95, 0.99]	0.0052
Stage				
3	reference	‐‐‐	reference	‐‐‐
4	4.34 [3.14, 6.01]	< 0.0001	5.45 [3.73, 7.96]	< 0.0001
Median household income				
16,683–61,508	reference	‐‐‐	reference	‐‐‐
61,509–83,767	0.47 [0.29, 0.75]	0.0017	0.57 [0.35, 0.93]	0.0232
83,768–233,173	1.22 [0.83, 1.79]	0.3175	1.74 [1.15, 2.65]	0.0092

^a^ Odds ratio and 95% Wald confidence interval from univariable logistic regression;

^b^ Adjusted odds ratio with 95% Wald confidence interval from multivariable logistic model containing covariates age, stage, and median household income.

A significant association between stage and referral for genetic counseling was observed in univariable analysis. The odds of referral were 4.34 times higher for patients with Stage IV disease when compared to patients with Stage III disease (OR = 4.34, 95% CI [3.14, 6.01], *p* < 0.0001). When controlling for age and gMHI, the odds of referral were 5.45 times higher for patients with Stage IV disease when compared to patients with Stage III disease (OR = 5.45, 95% CI [3.73, 7.96], *p* < 0.0001).

Lastly, there were significant associations between gMHI and referral for genetic counseling with patients in the middle‐income tertile less likely to be referred and patients in the highest‐income tertile more likely to be referred when compared to patients in the lower‐income tertile. The odds of referral were 53% times lower for patients in the middle‐income tertile when compared to patients in the low‐income tertile (OR = 0.47, 95% CI [0.29, 0.75]; *p* = 0.0017). Patients in the highest‐income tertile had higher odds of referral when compared to the lower‐income tertile, but did not reach a level of statistical significance (*p* = 0.3175). Similarly, when controlling for age and stage, odds of referral for patients in the middle‐income tertile were 43% lower (OR = 0.57, 95% CI [0.35, 0.93]; *p* = 0.0232) and the odds of referral were 1.74 times higher for patients in the high‐income tertile (OR = 1.74, 95% CI [0.35, 0.93]; *p* = 0.0092) when compared to patients in the low‐income tertile.

### 3.5 Probability of Predicting Completion of Genetic Testing Using Age, Stage and gMHI Among Stage IV Patients

Using univariable and multivariable logistic regression models, we assessed the efficacy of predicting completion of genetic testing by age, substage and gMHI (Table 5). No significant association between age and completion of testing (OR = 0.99, 95% CI [0.97, 1.01]; *p* = 0.2321) were observed for the unadjusted analysis. However, when controlling for substage and gMHI, there was a significant association between age and completion of testing. The odds of referral decreased by 3% for each 1‐year increase in age at diagnosis (OR = 0.97, 95% CI [0.95, 1.00]; *p* = 0.0218).

**Table 5:** Unadjusted and adjusted odds ratios predicting completion of genetic testing for stage IV patients.

	Unadjusted OR [95% CI]^a^	Unadjusted *p*‐value	Adjusted OR [95% CI]^b^	Adjusted *p*‐value
Age	0.99 [0.97, 1.01]	0.2321	0.97 [0.95, 1.00]	0.0218
Substage				
IVa	reference	‐‐‐	reference	‐‐‐
IVb	2.49 [1.60, 3.85]	< 0.0001	2.84 [1.70, 4.74]	< 0.0001
Median household income				
16,683–61,508	reference	‐‐‐	reference	‐‐‐
61,509–83,767	0.98 [0.56, 1.63]	0.9286	1.08 [0.64, 1.83]	0.7758
83,768–233,173	1.30 [0.77, 2.18]	0.3289	1.49 [0.87, 2.55]	0.1490

^a^ Odds ratio with 95% Wald confidence interval from univariable logistic regression;

^b^ Adjusted odds ratio with 95% Wald confidence interval from logistic model containing covariates age, substage, and median household income.

There was a significant association between substage and completion of genetic testing. The unadjusted analysis showed odds of completion of testing were 2.49 times higher for patients with substage IVb disease when compared to patients with substage IVa (OR = 2.49, 95% CI [1.60, 3.85]; *p* < 0.0001). When controlling for age and gMHI, the odds of completion were 2.84 times higher for patients with substage IVb when compared to patients with substage IVa (OR = 2.84, 95% CI [1.70, 4.74]; *p* < 0.0001).

There was no significant association between gMHI and completion of genetic testing for the unadjusted analysis as well when adjusted for age and substage.

## 4 Discussion

In this study, we found that the primary factors that affected referral for genetic counseling were age at diagnosis, race, stage, and MHI in univariable analysis. In multivariable analysis, referral for genetic counseling remained independently associated with age at diagnosis, stage, and gMHI. Subsequent completion of genetic testing, which depends on patient factors, was only associated with substage in univariable analysis and with substage, age, and gMHI in multivariable analysis.

Black patients were more likely to receive a referral for genetic counseling than White patients at our institution. This differs from some findings that have been previously reported across the country. A study published in 2023 found that germline testing rates for PCa patients in California were lower in Black patients than white patients (70% vs 85%, *n* = 403, OR 0.35, CI: 0.13–0.96) [10]. Genetic testing may be impacted by clinical factors rather than socioeconomics or race at our institution, making it an equal access institution as has been cited in other published studies [8]. Additionally, these associations with race were not independently associated when controlling for other factors.

Next, the stage was a significant association in both the univariable and multivariable analyses. We found that genetic testing completion, which is patient and not provider‐dependent, was more likely in patients with metastatic disease. These findings also align with previously published data that show a positive association between more advanced disease and genetic testing. Aguiar et al. found that utilization of genetic testing was positively associated with metastatic disease, speculating that this was due to its potential to impact treatment selection [4]. For patients with metastatic disease, substage IVb, germline genetic testing has a much higher utility, since treatments can be tailored to their specific cancer [5]. However, for patients with earlier stage disease, genetic testing is primarily useful for familial implications, but less critical in therapeutic regimen [11]. Additionally, not all variants identified in germline genetic testing are actionable, and some studies have cited that as few as 2% of findings may actually have targeted treatment options [12]. This drastic difference in utility not only affects patient interest and motivation to complete genetic testing, but also may change provider referral practices. Providers may be less likely to refer earlier‐stage patients to genetic testing because the need is less immediate, and the information may not contribute to treatment plans.

Additionally, gMHI was a significant association in the univariable analysis. Our findings indicate that high‐income and low‐income patients were more likely to receive a referral for genetic counseling than middle‐income patients. Evidence has shown that the expansion of Medicaid and the Affordable Care Act reduced the number of low‐income patients who did not have insurance. One study cited that the uninsured rate for patients below the Federal Poverty Line went from 8.4% to just 1.4% [13]. According to unpublished internal payer data for HFH obtained via trained abstraction from the HFCR, 59.5% of GU prostate cancer patients have Medicare, 7.1% have Medicaid and 29.4% of patients have private insurance (*n* = 452). This rise in health insurance for low‐income patients could account for their increased likelihood to receive a genetic counseling referral. Patients in the high‐income tertile are more likely to have private health insurance with broader coverage or pay for genetic testing out of pocket. This leaves the middle‐income tertile, who do not have access to the benefits of Medicaid and may be underinsured or more sensitive to cost. Education or awareness of resources could also be a contributor to these results. Based on research performed by the Social Security Administration in 2015, men with a bachelor's degree earn nearly $1 million more in median lifetime earnings than high school graduates [14]. This trend applies to women, with women with a bachelor's degree earning about $630,000 more [14]. Therefore, patients in the high‐income tertile are more likely to have a higher level of education, making them more poised to have deeper knowledge about genetic testing and better equipped to advocate for germline genetic testing themselves. Providers may also attempt to compensate for these inequities by spending more time educating and discussing genetic testing with low‐income patients, again leaving middle‐income patients overlooked. Despite these findings for referrals for genetic counseling, the gMHI association was not seen for completion of testing using multivariable analysis, indicating that the primary barrier to completion of testing may not be socioeconomic.

Lastly, no associations between SVI and referral for genetic counseling or completion of testing were seen. These findings indicate that patients who are more socially vulnerable were still able to access genetic testing and counseling services, despite facing more challenges than less socially vulnerable patients. Again, this may be due in part to the expansion of Medicaid and the ability of low‐income patients to access better health insurance [13].

One limitation of this analysis is that the data was only collected from a single institution, decreasing the generalizability of these results. As previously stated, demographics of the Detroit population differ significantly from the population in the United States at large. Providers may be more experienced in treating this specific population and therefore better equipped to help them overcome barriers to care.

Another limitation is that the data only incorporates referrals with a referral order in the electronic health record. Some providers may discuss genetic testing with their patients and after this discussion, choose not to enter a referral for genetic counseling. This could be due to a multitude of challenges including patient motivation, insurance coverage, disease management and others. The lower rates of referrals for certain groups, such as the middle‐income group or earlier stage patients, therefore, may reflect that groups' needs as shared with their provider in initial discussions.

## 5 Conclusion

This retrospective, single institution study examined demographic factors that are hypothesized to reduce PCa patients' utilization of genetic testing. Although race and socioeconomic factors have been thought to be barriers to testing, we found no evidence of such an association in our data. Instead, the lowest gMHI tertile patients were equally as likely to complete genetic testing as middle and highest gMHI tertile patients, indicating that if such barriers exist, they can be overcome. Further studies should be completed to assess these findings on a larger scale and to probe potential solutions to increase testing utilization for PCa patients.

## Author Contributions

Concept and design: Alexandra T. Skowron, Kyle C. McElyea, Avery K. Supernois, Clara Hwang. Collection and assembly of data: Alexandra T. Skowron, Kyle C. McElyea, Avery K. Supernois, Yousuf Hussain, Mohammed Baseer. Data analysis and interpretation: Alexandra T. Skowron, Courtney M. Rose, Karen Childers, Sunita Ghosh, Clara Hwang. Manuscript writing, final approval: All authors. Accountable for all aspects of the work: All authors.

## Conflicts of Interest

Dr. Hwang reported owning stock in Johnson and Johnson; receiving personal fees from Tempus, Genzyme, EMD Sorono, OncLive/MJH Life Sciences, Merck, and grants from Merck, Bausch Health, Genentech, Bayer, and AstraZeneca outside the submitted work.

## Data Availability Statement

The data that support the findings of this study are available from the corresponding author upon reasonable request.
